# Thalamus Atrophy in the Peri-Pregnancy Period in Clinically Stable Multiple Sclerosis Patients: Preliminary Results

**DOI:** 10.3390/brainsci11101270

**Published:** 2021-09-26

**Authors:** Iwona Rościszewska-Żukowska, Marek Podyma, Mariusz Stasiołek, Małgorzata Siger

**Affiliations:** 1 Neurology Outpatient Clinic, The Holy Family Specialist Hospital, Rudna Mala, 36-060 Rzeszow, Poland; rosciszewskaiwona1@gmail.com; 2 Medical College, University of Rzeszow, 35-310 Rzeszow, Poland; 3 Pixel Technology LLC, 93-558 Lodz, Poland; m.podyma@pixel.com.pl; 4 Department of Neurology, Medical University of Lodz, 22 Kopcinskiego Str, 90-153 Lodz, Poland; mariusz.stasiolek@umed.lodz.pl

**Keywords:** pregnancy, magnetic resonance imaging, multiple sclerosis, local brain atrophy, magnetic resonance imaging activity, peri-pregnancy period

## Abstract

Radiological activity in the post-partum period in MS patients is a well-known phenomenon, but there is no data concerning the influence of pregnancy on regional brain atrophy. The aim of this article was to investigate local brain atrophy in the peri-pregnancy period (PPP) in patients with MS. Thalamic volume (TV); corpus callosum volume (CCV) and classical MRI activity (new gadolinium enhancing lesions (Gd+), new T2 lesions, T1 lesions volume (T1LV) and T2 lesions volume (T2LV)) were analyzed in 12 clinically stable women with relapsing–remitting MS and with MRI performed in the PPP. We showed that there was a significant decrease in TV (*p* = 0.021) in the PPP. We also observed a significant increase in the T1 lesion volume (*p* = 0.028), new gadolinium-enhanced and new T2 lesions (in 46% and 77% of the scans, respectively) in the post-partum period. Our results suggest that the PPP in MS may be associated not only with classical MRI activity but, also, with regional brain atrophy.

## 1. Introduction

Magnetic resonance imaging (MRI) activity (new gadolinium-enhanced lesions (Gd+) and new/enlarged T2-weigheted lesions) in the post-partum period in multiple sclerosis (MS) patients has been demonstrated in a few published studies [1,2,3,4]. However, the impact of pregnancy on gray and white matter atrophy in MS is not well-characterized. In one study, no changes in brain parenchyma and cortical gray matter fraction were reported [4]. Thus, a protective role for pregnancy against brain volume loss has been suggested. However, to date, there are no data concerning the influence of pregnancy on thalamus and corpus callosum atrophy.

The aim of our study was to assess changes in the thalamic volume (TV) and corpus callosum volume (CCV), as well as in classical brain MRI activity (new Gd+ and new T2-weigheted lesions, T1 lesion volume (T1LV) and T2 lesion volume (T2LV)) associated with the peri-pregnancy period (PPP) in clinically stable women with MS.

## 2. Material and Methods

### 2.1. Patients

Twelve relapsing-remitting MS patients (RRMS) with a history of pregnancy during the disease were retrospectively identified in a local medical database. The patients were registered in the database between January 2015 and December 2019. Only clinically stable patients (no relapses 12 months before pregnancy, during pregnancy and in the 12-month post-partum period) who had a brain MRI with contrast administration at the PPP (defined as pre-pregnancy up to 6 months) and a follow-up (defined as the post-partum period up to 6 months) were included in the study. All of the patients had stable DMT treatment (INF beta1a i.m., *n* = 1; INF beta1b s.c., *n* = 6; glatiramer acetate, *n* = 3; dimethyl fumarate, *n* = 2) for at least 12 months before pregnancy. In each case, the treatment was stopped at the time of confirmed pregnancy. All the patients returned to their pre-pregnancy treatment after a follow-up MRI in the post-partum period. The neurological status was assessed by the Expanded Disability Status score (EDSS) [5] and a timed 25-ft walk (T25FW) at the baseline and follow-up.

This study was conducted according to the guidelines of the Declaration of Helsinki (1964) and its later amendments and approved by the Ethics Committee of the University of Rzeszow (protocol number 2/02/21). Informed consent was obtained from all the subjects involved in the study.

### 2.2. MRI Acquisition and Processing

Brain MRI was acquired on 1.5 T scanner (OPTIMA MR 360, GE HEALTHCARE, Pekin, China 2011) using a similar acquisition protocol performed within 3.1 ± 2.4 (mean ± *SD*, range 0.2–6.8) months before pregnancy and within 3.9 ± 3.5 (mean ± *SD*, range 0.5–12.3) months after delivery. There were no upgrades of software and hardware during the whole study period. The MRI protocol performed in all participants included the following sequences: T1-fast-spoiled gradient echo 3D (FSPGR 3D; TR = 5966 ms, TE = 1816 ms, flip angle = 12, slice thickness = 1.2 mm, number of slices = 144, Percent Phase FOV = 25 and matrix = 256 × 256); fast spin-echo-T2 (FSET2; TR = 5120, TE = 77.0, flip angle = 160, slice thickness = 3.0 mm, number of slices = 60, Percent Phase FOV = 25 and matrix = 256 × 256); T2-Fluid-Attenuated Inversion Recovery (T2-FLAIR; TR = 9000, ms TE = 80 ms, TI = 2250 ms, flip angel = 160, slice thickness = 3.0 mm, number of slices = 60, Percent Phase FOV = 25 and matrix = 256 × 192) and T1-weighted spin-echo (T1SE; TR = 500 ms, TE = 13 ms, flip angle = 80, slice thickness = 3.0 mm, number of slices = 60, Percent Phase FOV = 25 and matrix = 256 × 256). After gadolinium contrast administration (0.1 mmol/kg) and a delay of 10 min, the same T1SE sequence was acquired. All scans were visually inspected for quality.

The MRI lesions analysis was performed using a semi-automated, edge-finding tool in the Jim software package (v7.0, Xinapse System, West Bergholt, UK, http://www.xinapse.com (accessed on 1 December 2018), which is well-established and widely used throughout the neuroimaging MS research community. The analysis was done by one of the coauthors (MS) with >20 years of experience in this type of analysis. Left and right TV and CCV segmentation were performed using the automatic Exhibeon (v.3) software (https://www.allerad.com/en/dicom-viewer (accessed on 1 May 2020)) on the FSPGR 3D images. The segmentation module of Exhibeon software is based on 3D convolutional neural networks (CNN) trained on volumetric T1-weighted (with and without contrast enhancement) and T2-FLAIR MRI images [6]. The training dataset consisted of 2000 MRI studies from the OASIS-3 database [7] and 400 studies obtained as a part of the MRImmuno Project (see Funding). Brain structure segmentations, used as prediction labels in the CNN training set, were obtained using an automated pipeline of the FreeSurfer v6 software [8,9], as a well-established and widely tested brain MRI image processing and analyzing tool [10,11,12]. All FreeSurfer segmentations were executed with the default library settings (-all) and used as a CNN training set, without any manual correction. The CNN training process consisted of: image augmentation, denoising process (Total–Variation proximity), inhomogeneity bias field correction and data standardization (N4). All the regional segmentations results were reviewed and accepted by one of the coauthors (MS).The Exhibeon pipeline is specified in the Supplementary Materials.

### 2.3. Statistical Analysis

The variables are presented as the count *n* (% of group) for nominal variables or as the mean ± standard deviation (*SD*) or median and interquartile range (IQR), both with a range depending on distribution. The normality of distribution was validated using the Shapiro–Wilk test and based on a visual assessment of the histograms. The differences in TV, CCV, T1LV and T2LV between the pre-pregnancy and PPP scans were tested by a Wilcoxon signed-rank test. Changes were considered significant at the level of α = 0.05. A statistical analysis was carried out using StatSoft Statistica Analysis Software v9.0.

## 3. Results

**Figure 1:**
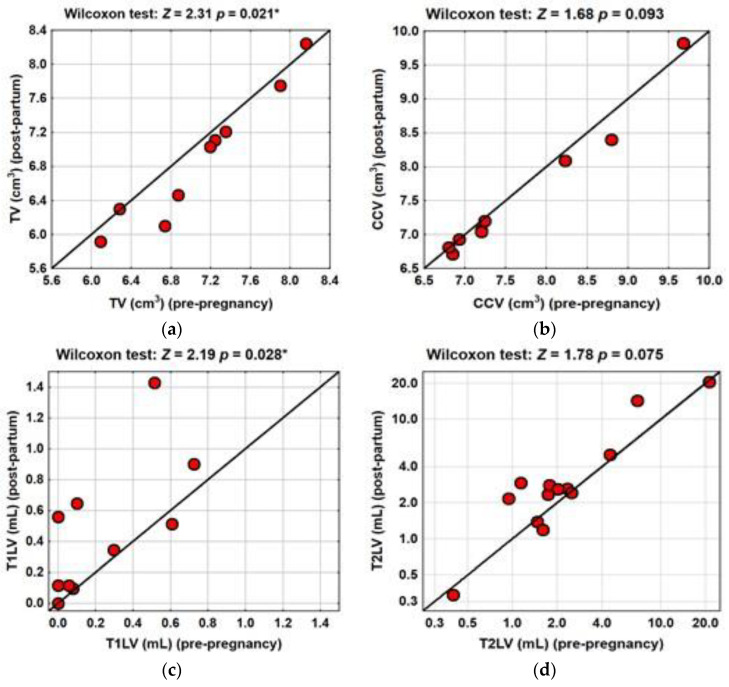
TV, CCV, T1LV and T2LV in the pre-pregnancy and post-partum MRI. Distribution of the median TV (**a**), CCV (**b**) T12LV (**c**) and T2LV (**d**) in the PPP. TV significantly decreased in the post-partum MRI compared with the pre-pregnancy period (*p* = 0.021). CCV did not differ in the PPP (*p* = 0.093). T1LV significantly increased in the post-partum MRI compared with the pre-pregnancy MRI (*p* = 0.028). T2LV increased (but not significantly) in the PPP (*p* = 0.075). Test statistics *Z*- and *p*-values were calculated using the Wilcoxon signed-rank test. A *p*-value was considered significant at the level of <0.05. Significant differences were indicated with (*).

The clinical and MRI characteristics of the study group in the PPP are presented in Table 1. In the PPP, we detected a significant decrease in TV (*p* = 0.021) (Table 1 and Figure 1a). CCV did not differ between the pre-pregnancy and post-partum MRI (*p* = 0.093) (Table 1 and Figure 1b). In the post-partum period, T1LV significantly increased compared with the pre-pregnancy scans (*p* = 0.028), but T2LV was not significantly different in the PPP (*p* = 0.075) (Table 1 and Figure 1c,d, respectively). Additionally, new Gd+ lesions were detected in 6/13 (46.2%) scans, and new T2-weighted lesions were observed in 10/13 (76.9%) scans.

## 4. Discussion

Our results demonstrated TV loss and changes in classical MRI parameters in the PPP in clinically stable women with MS.

The knowledge concerning brain MRI atrophy measures in patients with MS during and after pregnancy remains very limited. In one study by Khalid and colleagues [4], the authors did not find any statistically significant differences in brain parenchyma and cortical gray matter fractions between the pre-pregnancy and post-partum periods. In another study [13], the whole brain volume and ventricular size were assessed during pregnancy and after delivery in healthy volunteers and in patients with preeclampsia. This study revealed a special pattern of brain and ventricular volume changes during pregnancy and after delivery. The authors explained the observed changes as a result of hormonal and metabolic alterations [13]. However, little is known about other atrophy measures in this clinical context.

Several explanations for the TV loss observed in our MS patients can be suggested. Based on the previous published results [14], we can speculate that TV loss in our patients may result from axonal transection within the white matter lesions located in the tracts going to and from the thalamus, followed by the upstream and downstream degeneration of this tract. We can also assume that thalamic atrophy may be the consequence of iron deposition, a process that has been described previously in patients with MS [15]. However, our MRI protocol did not include sequences required to detect iron deposition. Finally, we cannot exclude the microstructural changes in thalamus gray matter as contributing to TV loss [16]. Interestingly, in our group, a decrease in TV in the PPP was observed in clinically stable women. We suggest that it may be associated with the ongoing process of neurodegeneration presenting as a local brain volume loss, despite an apparent clinical stability.

In our study we did not find differences in CCV between the pre-pregnancy and post-partum periods. Corpus callosum represents a white matter structure, very specific for focal lesion locations in MS [17]. However, a decrease of CCV in MS has not always been shown to proceed parallel to a gray matter volume loss [18,19]. Thus, based on previous observations, we can speculate that TV and CCV changes may progress independently during the course of MS [11,12].

In our group, we found a significant increase in T1LV in post-partum MRI. Lesions on T1-weighted images (“black holes”) represent area of severe tissue damage, including axonal loss, irreversible demyelination and matrix destruction [20]. The increase of the T1 lesion volume is considered a very sensitive MRI predictor of disease progression [20]. Our finding of a significant increase of T1LV in the PPP is in-line with the previously published result by Khalid et al. [4], who also showed a statistically significant increase of T1LV in the post-partum period. We can speculate that hormonal and immunological changes during pregnancy and soon after delivery may contribute to the process of brain neurodegeneration and to the increase of T1LV, even without overt clinical activity. However, we did not detect significant changes in T2LV in the PPP, which is not concordant with the previously published data [1,2,4]. We speculate that the small sample size of our group may have contributed to this result.

However, classical MRI activity on post-partum scans was detected in most of our patients. Our finding concerning new GD+ and new T2 lesions in the post-partum period is consistent with previously published studies [1,2,3,4]. We are aware that the classical MRI activity observed in our patients may be related to the discontinuation of DMT, but on the other hand, the results from published research are not consistent regarding this issue [3,4,21].

This study was not without limitations. The main limitations of our study were the retrospective study character, the small sample size and the lack of a control group (healthy pregnant women with MRI pre- and post-delivery). Another limitation was the relatively short period of time between the pregnancy and post-partum MRI. However, this study was based on the results obtained in a group of women with MS with a history of pregnancy that were followed clinically and radiologically according to the standardized procedures in a single MS center. We find it of great importance to follow our group of patients. Accordingly, we plan to document and analyze future developments of both their clinical and MRI findings and, also, compare the results with a new group of MS patients treated during pregnancy.

## 5. Conclusions

Our preliminary results showed that the influence of pregnancy on the MS course is very complex. Even in clinically stable patients, the PPP may be associated with both local brain atrophy and classical MRI activity. We think that our findings deserve further investigation as a possible source of relevant clinical information in the assessment of the therapy effectiveness in women with MS undergoing pregnancy.

## Figures and Tables

**Table 1:** Patient demographic, clinical and MRI characteristics.

	Pre-Pregnancy	Post-Partum	Test Statistics	*p*-Value
Number of patients, *n*	12	12		
Age (years), mean ± *SD* (range) ^a^	31.4 ± 3.4			
	(26.7 to 38.0)			
Weight (kilogram), mean ± *SD* (range)	63 ± 9.98	65 ± 7.8		0.938
	(58.8 to 72.5)	(61.4 to 68.2)		
Disease duration (years), mean ± *SD* (range) ^b^	5.7 ± 4.9			
	(1.1 to 14.9)			
Time of MRI (months), mean ± *SD* (range) ^c,d^	3.1 ± 2.4	3.9 ± 3.5		
	(0.2 to 6.8) ^c^	(0.5 to 12.3) ^d^		
EDSS score, median (IQR) (range)	1.5 (1.0)	1.5 (0.5)	*Z* = 0.89	0.371
	(1.0 to 4.0)	(1.0 to 5.0)		
T25FW (seconds), mean ± *SD* (range)	4.40 ± 0.64	4.52 ± 0.88	*Z* = 0.08	>0.999
	(3.33 to 5.13)	(3.30 to 5.60)		
MRI activity				
TV (cm^3^), median (IQR) (range)	7.04 (1.25)	6.74 (1.25)	*Z* = 2.31	0.021 *
	(5.90 to 8.16)	(5.92 to 8.2)		
CCV (cm^3^), median (IQR) (range)	7.20 (1.54)	7.05 (1.33)	*Z* = 1.68	0.093
	(6.15 to 9.68)	(6.71 to 9.82)		
T1LV (mL), median (IQR) (range)	0.09 (0.46)	0.34 (0.55)	*Z* = 2.19	0.028 *
	(0.00 to 0.7)	(0.00 to 1.43)		
T2LV (mL), median (IQR) (range)	1.78 (2.22)	2.60 (2.21)	*Z* = 1.78	0.075
	(0.40 to 21.22)	(0.34 to 20.47)		
Number of scans with new Gd+ lesions (across all pregnancies), *n* (%)		6/13 ^e^ (46.2)		
Number of scans with new T2 lesions (across all pregnancies), *n* (%)		10/13 ^e^ (76.9)		

Abbreviations: *n*—number. *SD*—standard deviation; IQR—interquartile range; MR—magnetic resonance imaging; EDSS—Expanded Disability Status Score; T25FW—timed 25-ft walk; TV—thalamic volume; CCV—corpus callosum volume; T1LV—T1 lesion volume; T2LV—T2 lesion volume.; new Gd+ lesions—lesions detected on T1-derived sequences after gadolinium contrast administration in the post-partum MRI not visible on the baseline scans; new T2 lesions—lesions detected on T2-derived sequences in the post-partum MRI but not visible on the baseline scans; ^a^ age at onset of pregnancy; ^b^ duration of disease at onset of pregnancy; ^c^ time between the pre-pregnancy MRI and pregnancy; ^d^ time between the delivery and post-partum MRI; ^e^ number of pregnancies. Test statistics *Z*- and *p*-values were calculated using the Wilcoxon signed-rank test; a *p*-value was considered significant at the level of <0.05. * Indicates significant differences between the pre- and post-partum volumes.

## Data Availability

The data presented in this study are available on request from the corresponding author. The data are not publicly available due to the local ethical regulations.

## Supplementary Materials

The following are available online at https://www.mdpi.com/article/10.3390/brainsci11101270/s1: 1. Exhibeon 3 pipeline.
