# Supplementary material for: Thalamus Atrophy in the Peri-Pregnancy Period in Clinically Stable Multiple Sclerosis Patients: Preliminary Results
Source: Brain Sci. 2021 Sep 26;11(10):1270. doi: 10.3390/brainsci11101270 (PMC8534211; doi:10.3390/brainsci11101270)
Supplement: Supplementary file 1 [file brainsci-11-01270-s001.zip › brainsci-1286818-supplementary.pdf]

We certify that the following article

Thalamus atrophy in peri-pregnancy period in clinically stable multiple sclerosis patients

Malgorzata Siger

has undergone English language editing by MDPI. The text has been checked for correct use of grammar and common technical terms, and edited to a level suitable for reporting research in a scholarly journal.
MDPI uses experienced, native English speaking editors. Full details of the editing service can be found at
► https://www.mdpi.com/authors/english.

MDPI Multidisciplinary Digital Publishing Institute

Basel, Switzerland
May 2021

AUTHORservices

Exhibeon 3 pipeline

Brain structures were automatically segmented using Exhibeon 3 (*Pixel Technology LLC, Poland*), a medical imaging software (CE cleared) with integrated brain anatomical segmentation toolbox (https://www.allerad.com/en/dicom-viewer). Segmentation mechanism is based on 3D convolutional neural networks (CNN) trained on volumetric T1-weighted (with and without contrast enhance) and T2-FLAIR MRI images [1]. T2-FLAIR series was treated as nonobligatory, consequently CNN topology and optimization methods were designed to randomly omit this sequence during both training and inference processes.

Training dataset was based on 2000 MRI studies from the OASIS-3 database and 400 studies obtained as a part of the MRImmuno project [2]. Brain structures segmentations, used as prediction labels in CNN training set, were obtained using an automated pipeline [3,4] - FreeSurfer v6, as well established and widely tested brain MRI images processing and analyzing tool [5-7]. All FreeSurfer segmentations were executed with default library settings (--all) and used in CNN training without any manual correction. CNN training process consisted of: image augmentation, denoising process based on Total Variation proximity[8,9], inhomogeneity bias field correction and data standardization based on N4 filtering from ITK package [10].

Quality controls were performed after each processing step and systematically reviewed. Thalami and corpus callosum (CC) labels acquired from CNN model were reviewed and manually corrected by experienced rater, blinded to clinical data, using semi automated labeling tools built in Exhibeon3.

Briefly, the overall method implemented, consisted of following steps: (1) Execution of FreeSurfer segmentation for each of 2400 T1-weighted series with optional T2 FLAIR (if available); (2) execution of CNN network training process consisting of: data augmentation, standardization, denoising and inhomogeneity bias field correction. (3) Automatic segmentation using Exhibeon 3 integrated with CNN, verification and manual correction of thalami and CC volumes [8,10,11,12,13].

References

1. Bontempi D, Benini S, Signoroni A, Svanera M, Muckli L. CEREBRUM: a fast and fully-volumetric Convolutional Encoder-decodeR for weakly-supervised sEgmentation of BRain strUctures from out-of-the-scanner MRI. Med Image Anal. 2020 May;62:101688. DOI: 10.1016/j.media.2020.101688. Epub 2020 Mar 24. PMID: 32272345.
2. Marcus DS, Fotenos AF, Csernansky JG, Morris JC, Buckner RL. Open access series of imaging studies: longitudinal MRI data in nondemented and demented older adults. J Cogn Neurosci. 2010 Dec;22(12):2677-84. DOI: 10.1162/jocn.2009.21407. PMID: 19929323; PMCID: PMC2895005.
3. Ronneberger O., Fischer P., Brox T. (2015) U-Net: Convolutional Networks for Biomedical Image Segmentation. In: Navab N., Hornegger J., Wells W., Frangi A. (eds) Medical Image Computing and Computer-Assisted Intervention – MICCAI 2015. MICCAI 2015. Lecture

Notes in Computer Science, vol 9351. Springer, Cham. DOI: 10.1007/978-3-319-24574-4_28
4. Guha Roy A, Conjeti S, Navab N, Wachinger C; Alzheimer's Disease Neuroimaging Initiative. QuickNAT: A fully convolutional network for quick and accurate segmentation of neuroanatomy. Neuroimage. 2019 Feb 1;186:713-727. DOI: 10.1016/j.neuroimage.2018.11.042. Epub 2018 Nov 29. PMID: 30502445.
5. Fischl B. FreeSurfer. Neuroimage. 2012 Aug 15;62(2):774-81. DOI: 10.1016/j.neuroimage.2012.01.021. Epub 2012 Jan 10. PMID: 22248573; PMCID: PMC3685476.
6. Burggraaff J, Liu Y, Prieto JC, Simoes J, de Sitter A, Ruggieri S, Brouwer I, Lissenberg-Witte BI, Rocca MA, Valsasina P, Ropele S, Gasperini C, Gallo A, Pareto D, Sastre-Garriga J, Enzinger C, Filippi M, De Stefano N, Ciccarelli O, Hulst HE, Wattjes MP, Barkhof F, Uitdehaag BMJ, Vrenken H, Guttmann CRG; MAGNIMS Study Group. Manual and automated tissue segmentation confirm the impact of thalamus atrophy on cognition in multiple sclerosis: A multicenter study. Neuroimage Clin. 2021;29:102549. DOI: 10.1016/j.nicl.2020.102549. Epub 2020 Dec 25. PMID: 33401136; PMCID: PMC7787946.
7. Koskimäki F, Bernard J, Yong J, Arndt N, Carroll T, Lee SK, Reder AT, Javed A. Gray matter atrophy in multiple sclerosis despite clinical and lesion stability during natalizumab treatment. PLoS One. 2018 Dec 21;13(12):e0209326. DOI: 10.1371/journal.pone.0209326. PMID: 30576361; PMCID: PMC6303064.
8. Valverde S, Cabezas M, Roura E, González-Villà S, Pareto D, Vilanova JC, Ramió-Torrentà L, Rovira À, Oliver A, Lladó X. Improving automated multiple sclerosis lesion segmentation with a cascaded 3D convolutional neural network approach. Neuroimage. 2017 Jul 15;155:159-168. DOI: 10.1016/j.neuroimage.2017.04.034. Epub 2017 Apr 19. PMID: 28435096.
9. Alvaro Barbero, & Suvrit Sra (2018). Modular Proximal Optimization for Multidimensional Total-Variation Regularization. Journal of Machine Learning Research, 19(56), 1-82.
10. Tustison, N. & Gee, J. (2009). N4ITK: Nick's N3 ITK implementation for MRI bias field correction. Insight J. 1-8.
11. Sled JG, Zijdenbos AP, Evans AC. A nonparametric method for automatic correction of intensity nonuniformity in MRI data. IEEE Trans Med Imaging. 1998 Feb;17(1):87-97. DOI: 10.1109/42.668698. PMID: 9617910.
12. Avants BB, Tustison NJ, Wu J, Cook PA, Gee JC. An open source multivariate framework for n-tissue segmentation with evaluation on public data. Neuroinformatics. 2011 Dec;9(4):381-400. DOI: 10.1007/s12021-011-9109-y. PMID: 21373993; PMCID: PMC3297199.
13. Lehtinen, J., Munkberg, J., Hasselgren, J., Laine, S., Karras, T., Aittala, M., & Aila, T. (2018). Noise2Noise: Learning Image Restoration without Clean Data. In Proceedings of the 35th International Conference on Machine Learning (pp. 2965–2974). PMLR.
